# Lightweight and Durable PVDF–SSPF Composites for Photovoltaics Backsheet Applications: Thermal, Optical and Technical Properties

**DOI:** 10.3390/ma12132104

**Published:** 2019-06-29

**Authors:** M. H. Alaaeddin, S. M. Sapuan, M. Y. M. Zuhri, E. S. Zainudin, Faris M. AL- Oqla

**Affiliations:** 1 Advanced Engineering Materials and Composites Research Center, Department of Mechanical and Manufacturing Engineering, Faculty of Engineering, Universiti Putra Malaysia, Serdang 43400 UPM, Selangor, Malaysia; 2 Laboratory of Bio-Composite Technology, Institute of Tropical Forestry and Forest Products, Universiti Putra Malaysia, Serdang 43400 UPM, Selangor, Malaysia; 3 Department of Mechanical Engineering, Faculty of Engineering, The Hashemite University, 13133 Zarqa, Jordan

**Keywords:** photovoltaic applications, backsheets, natural fiber composites, optical properties, thermal properties, technical properties

## Abstract

Photovoltaic module backsheets are characterized according to their thermal, optical, mechanical, and technical properties. This work introduces new fabricated backsheets for PV modules using polyvinylidene fluoride (PVDF) reinforced with short sugar palm fiber (SSPF) composites. The preparation of composites undergoes multiple phases of fabrication. Thermal, optical, and technical investigations of their properties were conducted. Fourier transform infrared spectroscopy (FTIR), Raman spectroscopy, in-situ scanning probe microscopy (SPM), dynamic mechanical analysis (DMA), thermal mechanical analysis (TMA), thermogravimetric analysis (TGA), differential scanning calorimetry (DSC), and prolonged technical testing were accomplished to expansively understand the complex behavior of composites under various conditions. The optical properties of PV backsheets are critical components in determining the reflectance, absorbance, and transmittance of light. The PVDF–SSPF composites exhibited exceptional compatibility and thermal stability, further revealing a homogenous composite structure with enhanced interfacial bonding between the short fiber and polymer matrix.

## 1. Introduction

Photovoltaic (PV) module backsheets are essential structural components that influence the overall performance; they provide strength and enhance durability [1,2]. With various multilayer backsheet structures, commercial backsheets are characterized in accordance with their basic optical and mechanical properties as well as their composite structure. These are based on the main constituents and multilayer laminates composed of inorganic modifiers and various polymeric materials. Such multilayer structures influence the various key features of PV backsheets (i.e., optical, thermal, electrical, mechanical, and barrier properties) to the specific requirements of PV modules [3].

The temperature control in a PV module has been recently identified as another vital factor that affects the overall efficiency of energy conversion, it exponentially influences the performance of solar cells. PV modules are constantly exposed to the natural variability of stress factors that occur in outdoor environments. To reduce increasing operating temperature, it is crucial to maintain high stability of the mechanical, chemical, and electrical properties of PV backsheets for long-term safety and reliability [4]. 

Natural fiber (NF) materials are succeeding in various composites and engineering applications. NFs have been introduced as competitive alternatives for synthetic fibers and are employed in countless products [5,6]. This is due to their outstanding chemical, physical, thermal, environmental, and mechanical characteristics in which the selected type of fiber can be applied as reinforcement in polymers. They are mostly applied as fillers for polymer-based matrices. The concept of natural fiber composites (NFCs) is crucial for material composites and engineered products [7,8]. The utilization of natural “fibers” has been recently considered for fiber composite-based materials. Such fibers are extracted from sisal, jute, hemp, kenaf, flax, coir, sugar palm, date palm, oil palm, abaca, ramie, plants leaves, henequen leaves, etc. [9,10]. 

The introduction of new backsheet structures and composite laminates may contribute to the efficiency of PV modules. They can enhance their adequacy, durability, and functional properties, e.g., moisture-proofing, reflectance, heat dissipation, thickness swelling, degradation, durability, protection, etc. [11,12]. Most PV backsheet composites employ various engineering thermoplastics and materials for indoor and outdoor applications, e.g., fluoropolymers, polyvinyl fluoride (PVF) known as Tedlar, polyvinylidene fluoride (PVDF) used as an isolative material to retain strength and durability and can be applied in inner and outer layers, ethyl vinyl acetate (EVA) which likewise functions as an encapsulant that strengthens the mechanical properties and enhances bonding between solar cells and backsheets in PV modules, as well as polyamides (PA) sheets and polyethylene terephthalate (PET) composite films which are also used as outer layers of protective material for PV backsheets [13,14,15,16,17]. 

The novelty of this work is in developing biocomposite materials based on natural fiber composites, to investigate their properties as potential components for PV backsheets. The integration between PVDF (which provides excellent weatherability and exhibits great mechanical stability) with SSPF (which displays outstanding physical and reinforcement properties) is believed to establish new opportunities that will positively contribute to the overall performance of PV modules.

## 2. Materials and Methods 

The composites consist of two main materials: Sugar palm fiber (SPF) extracted from matured sugar palms located at Kuala Jempol, Negeri Sembilan, Malaysia, and polyvinylidene fluoride (PVDF) purchased in pellet form. PVDF was obtained from a manufacturer in China. Both SPF and PVDF were processed and fabricated in the laboratories of Universiti Putra Malaysia (UPM). The composites’ fabrication process, as well as the optical, thermal, and technical testing, are discussed in this section.

### 2.1. Fabrication of Composites

The obtained fiber was prepared and made into short fiber through sequential phases: (1) The fiber threads were manually picked according to their purity, length, and strength, (2) the selected threads underwent a crushing phase and were cut into small pieces using a crushing machine, (3) the crushed fibers further endured a grinding process, finally, (4) the fibers were refined and sieved using a 200–250 micron sieve analyzer. The obtained short sugar palm fibers (SSPFs) were naturally dried and prepared for the mixing process. The SSPFs and PVDF were mixed using a Brabender measuring mixer, model: W 50 EHT (Brabender, Duisburg, Germany). As recommended in several studies, the loading of fiber in composites was 30%. This percentage ensures better mechanical, environmental, and technical properties such as hardness, tensile strength, flexural strength, impact strength, enhanced interfacial bonding, abrasion resistance, and thermal stability. Such enhanced properties will provide adequate reinforcement in composites [18,19,20,21]. The composites then underwent a hot and cold press accomplished through successive phases. First, the specimens experienced a preheat of 10 min, hot-pressed for five minutes, and then cold-pressed for 1 min at a maximum pressure of 160 Bar. The used mold measurement was 150 mm (L) × 150 mm (W) × 2 mm (T). 

### 2.2. Optical Testing of Composites

The Fourier transform infrared spectroscopy (FTIR), Raman spectroscopy, and in-situ scanning probe microscopy (SPM) were conducted to investigate the chemical absorption and bonding properties of the composites. Infrared and Raman spectroscopies are significant methods that provide additional analyses and information on the chemical nature of employed materials. The tests contribute to the investigation of surface composition as well as the interactions developed within the composites [22]. In-situ scanning probe microscopy (SPM) was accomplished to extensively comprehend the nanostructure and surface topography of the specimens. The FTIR test was carried out using the Thermo Scientific Model: Nicolet 6700 (Thermo Electron, Madison, USA) and applying the attenuated total reflection technique. The Raman spectroscopy was conducted using WITec Raman microscope model: Alpha 300R (WITec, Ulm, Germany) and the single spectrum analysis was employed. The SPM images were obtained from the nanomechanical testing machine, Hysitron TI950 Triboindenter (Bruker, Massachusetts, USA). Nanomechanical testing is a key technique for characterizing composites and evaluating their behavior at the smallest possible scale.

### 2.3. Thermal Testing of Composites

Four main tests were included in the thermal testing of composites: The dynamic mechanical analysis (DMA) using a dynamic mechanical analyzer (Model: Q800, TA Instruments, New Castle, USA), thermal mechanical analysis (TMA) using a thermal mechanical analyzer (Model: Q400, TA Instruments, New Castle, USA), thermogravimetric analysis (TGA) using a thermal gravimetric analyzer (Model: Q500, TA Instruments, New Castle, USA), and differential scanning calorimetry (DSC) using a differential scanning calorimeter (Model: Q20, TA Instruments, New Castle, USA). Most composite materials (especially those considered with temperature dependent materials or applications) require excessive thermal examinations of their properties. High temperatures may cause system deficiency in the PV module. Therefore, developing an effective method to enhance solar cell efficiency by cooling the PV cells or at least preventing the solar module from gaining excessive heat will contribute to their overall efficiency. This may involve the utilization of photovoltaic thermal-phase change material systems or by employing new innovative methods which combine natural fiber materials with the component or structure of the PV module. For example, using natural fiber composites and their distinctive properties to prevent solar cells from experiencing excessive heat [16]. The FTIR and Raman spectroscopies are essential tests conducted in various studies regarding photovoltaic backsheets. They reveal the composites’ optical properties and identify the polymeric materials of various structures [3].

### 2.4. Technical Testing of Composites

Three main tests were performed to investigate the composites’ technical properties: Prolonged testing method (applied for the moisture content), water absorption, and thickness swelling. The prolonged testing is necessary to determine the important properties of composites fabricated for outdoor applications since they can still be influenced by moisture uptake, fungal resistance, thermal stability, and ultraviolet (UV) stability. Therefore, it is essential to improve the lifespan of natural fiber composites according to their applications [23].

To measure the moisture content, five specimens were employed. Each weighed 2.0 g. An average value was obtained using the UniBloc moisture analyzer MOC63u (Shimadzu Corp., Kyoto, Japan). In the water absorption test, another five specimens were also prepared and artificially dried using (PROTECH Gov-100, Tech-Lab, Selangor, Malaysia) natural/gravity convection oven for 24 h. Prolonged immersions for 300, 1000, 1500, and 2000 h were accomplished. The water absorption test was also included in this section to ensure reliability and to analyze the behaviors of composites in long durations of water immersion. In each process, specimens were weighed before and after being immersed in distilled water and were then kept at room temperature (27 ± 0.5 °C). In the calculation process, water absorption was determined as:

(1) $$Wa=\;\frac{W_{i}-\;W_{0}}{W_{0}}\times 100\%$$

where Wa is the water absorption, $W_{i}$ is the weight of each specimen after immersion, and $W_{0}$ is the weight of each specimen before immersion. 

The thickness swelling test was accomplished using the Digital Vernier, model (Absolute Digimatic Caliper: Mitutoyo, Kawasaki, Japan). A prolonged testing method was conducted for 1500 h. The prolonged testing of composites can be assessed by the very low water absorption observed. Therefore, short-duration immersion may not display any remarkable results. The specimens’ dimensions were 20 mm (L) × 10 mm (W) × 2 mm (T). As a result, three-point measurement was taken for each specimen. An average value was calculated for each test. The thickness swelling was measured using the following equation:

(2) $$Ts=\;\frac{T_{i}-\;T_{0}}{T_{0}}\times 100\%$$

where Ts is the thickness swelling, $T_{i}$ is the weight of each specimen after immersion in distilled water, and $T_{0}$ is the weight of each specimen before the immersion process.

## 3. Results and Discussion

### 3.1. Optical Properties

#### 3.1.1. Fourier Transform Infrared Spectroscopy (FTIR)

**Figure 1:**
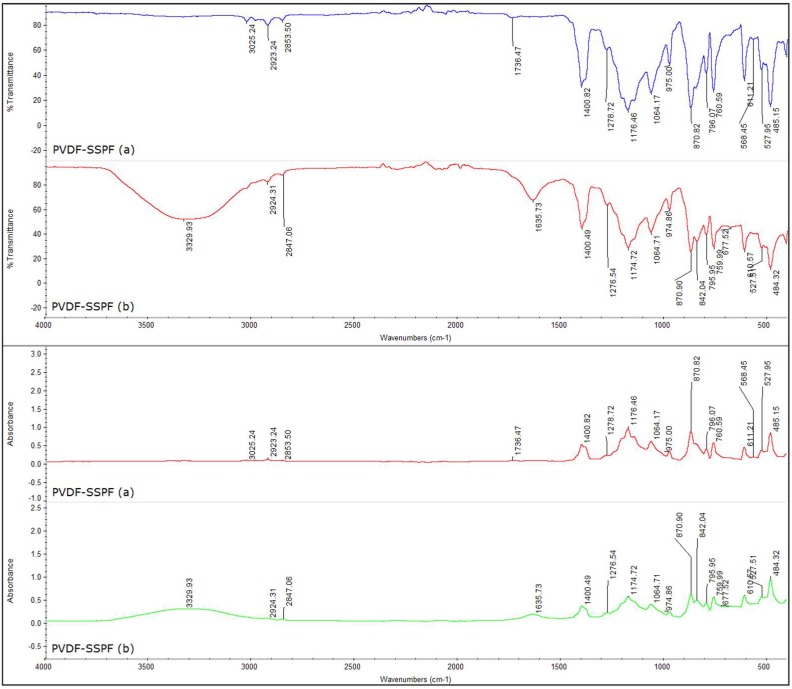
FTIR spectra of the PVDF–SSPF composites with wavenumber ranges from 4000 cm^−1^ to 500 cm^−1^, (**a**) non-immersed specimens, (**b**) immersed specimens.

Figure 1 presents the FTIR spectra for PVDF–SSPF composites. Normal and 2000-hour immersed specimens were investigated in the FTIR test to assess and characterize the chemical bonds in the matrix composites and to determine the main absorption regions.

The FTIR spectra of PVDF–SSPF composites provide additional information on the characterization of chemical bonds in the fiber matrix. As shown in Figure 1, exposing the specimens to water leads to slight changes in the molecular interactions. A shift in wavenumber can be observed. The wavenumber shifts in the FTIR process ranged between 4000 cm^−1^ and 500 cm^−1^. For non-immersed specimens, the wavenumber shifts occurred at approximately 3025.24 cm^−1^, 2923.24 cm^−1^, 2853.50 cm^−1^, and 1736.47 cm^−1^, respectively. Intense peaks in wavelength occurred between 1400.82 cm^−1^ and 485.15 cm^−1^. For the immersed specimens, the major wavenumber shifts occurred at 3329.93 cm^−1^, 2924.31 cm^−1^, 2847.06 cm^−1^, and 1635.73 cm^−1^. Intense peaks were also observed between 1400.49 cm^−1^ and 484.32 cm^−1^. However, the broadband appeared between 3000 cm^−1^ and 3500 cm^−1^ which may be assigned to the hydrogen bonded hydroxyl group (O‒H) derived from the complex vibrational stretching linked to the molecular bond of hydroxyl groups [24]. The band ranged between 2923.24 cm^−1^ and 2924.31 cm^−1^. The bands of 2853.50 cm^−1^ and 2847.06 cm^−1^ were anticipated to link to the C‒H stretching vibration from CH_2_ and/or CH_3_ [24,25]. On the other hand, peaks ranging between 1736.47 and 1635.73 can be assigned to the C=O stretching. This may represent the spectrum of SPF, as proven by [26]. However, the FTIR analysis only provides information on the wavenumbers of transmittance values (%) and absorbance by specimens. Immersed specimens exhibited slightly higher instability due to the major shifts in wavenumbers. This may be due to the insignificant changes of composition as well as the fiber matrix response to prolonged water immersion. Slight changes in the transformation of crystalline structure can also be the case, as reported by [27]. Result variations can be considered insignificant. Regarding the fiber, its relative absorbance which represents the amount of various functional groups remained the same despite shifting heights. The mechanical properties of SPF maintained adequate correlation concerning their chemical compositions. Hence, the fiber’s hemicellulose, cellulose, and lignin provide a significant contribution to their mechanical bonds [28]. A high resemblance can be seen in both analyses regarding the acquired peaks, such as 2923.24 cm^−1^ and 2924.31 cm^−1^ as well as 2853.50 cm^−1^ and 2847.06 cm^−1^. Only a slight insignificant variation was present due to water absorbance in specimens (b). Intense peaks with increased likeness in both charts have been observed, ranging from 1400.82 cm^−1^ and reaching 485.15 cm^−1^ in the non-immersed specimens and 1400.49 to 484.32 cm^−1^ in the immersed specimens. The peak at 3329.93 cm^−1^ in the immersed specimens may be assigned to adsorbed water since they were kept in water for 2000 h, as supported by [26]. Overall, the FTIR analysis demonstrated the existence of specific chemical constituents in the PVDF–SSPF composites. Since the used fiber matrix is derived from biological resources, the FTIR spectra exhibited nearly the same peaks. This mainly represents the exceptional stability in absorbance and transmittance properties, as further supported by [29].

#### 3.1.2. Raman Spectrum

**Figure 2:**
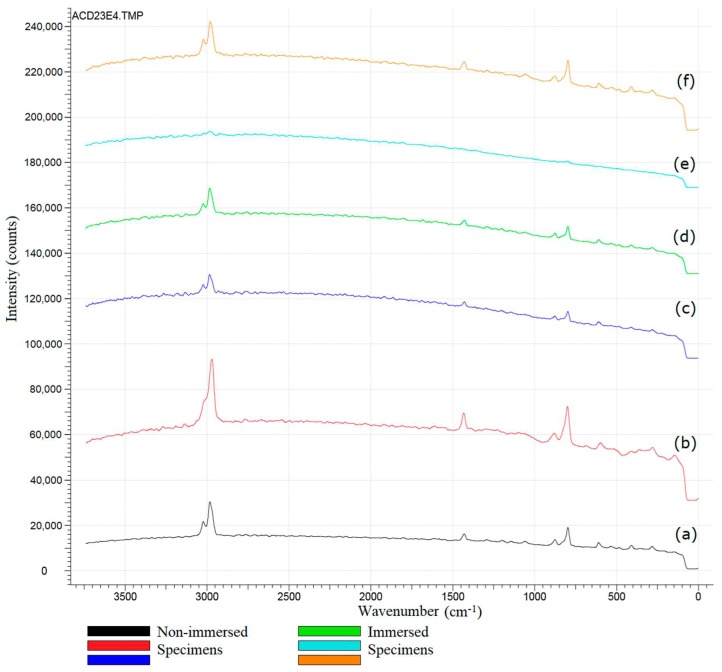
Representative raw Raman spectra of PVDF–SSPF composites. (**a**–**c**) Spectra of the non-immersed specimens. (**d**–**f**) Spectra of the immersed specimens.

The novel characterization of PVDF–SSPF composites by Raman spectroscopy provides further evidence on the composites’ overall performance. It has been employed as an effective experimental method to delicately probe the non-covalent interactions with various molecular moieties. It is recommended to characterize the structural changes of composite materials in which a contribution to the classification of content can be maintained. This is accomplished by understanding the behavior of materials that contain hexagonally packed aromatic hydrocarbons via the stretching vibrations of C‒C bonds in the aromatic rings [30]. Raman and infrared spectroscopies can provide “complementary” analyses on the molecular vibrations in composite materials with advances in chemical mapping [31,32]. Figure 2 provides representative raw Raman spectra of PVDF–SSPF composites. 

In the process of Raman spectroscopy, the excitation wavelength was applied through 531.747 nm, grating T1: 600 g/mm BLZ = 500 nm, and center wavelength of 595.222 nm. The vertical shift speed was 16.25 µs and the horizontal shift speed was 0.033 MHz. The characteristic Raman peaks for PVDF–SSPF have been shown and repeated on three different sites in both types of specimens. Not all signals can be easily attributed to specific group frequencies in both the FTIR and Raman tests. In the given results, the initial analysis of data indicated that the SSPF is characterized by specific bands ranging between 200 cm^−1^ and 1700 cm^−1^. The PVDF is likely characterized by the major bands ranging between 2000 cm^−1^ and 3400 cm^−1^. Raman spectroscopy supports differentiating fibers based on their composition. This result is also maintained in other studies [33]. Additionally, the Raman shifts observed in the given results indicate that signals ranging between 600 cm^−1^ and 1700 cm^−1^ can be assigned to conjugated C=O or C=C stretching modes. Signals ranging between 800 cm^−1^ and 1000 cm^−1^ can be designated to the C=N stretching mode of the composites. However, the Raman spectrum peak for PVDF–SSPF of both specimens ranged between 2950 cm^−1^ and 3000 cm^−1^. The peak corresponds to the ring-stretching modes of the cellulose structure [34] or composite interactions as possible anticipation. Alternatively, the smaller peaks that appeared between 900 cm^−1^ and 1200 cm^−1^ are more likely to represent the C‒O‒C stretching vibrations from carbohydrates and C‒C ring-breathing, this was justified by [31,32]. The most prominent bands are likely to appear in the range of 2900 cm^−1^ and 3100 cm^−1^. These peaks can be accredited to O–CH_3_, C–CH_3_, and CH_2_ stretching modes [32]. Additionally, minimized peaks were observed in the Raman spectra of immersed specimens, especially in specimen (e). This was due to the water content in composites which made peaks appear shorter than those of non-immersed specimens. Both shifts provide strong evidence that the fiber did interact with the polymer matrix. However, numerous studies have investigated the polymer-fiber composites using Raman spectroscopic techniques, including diverse types of polymers and fibers. Some tests, however, relied on numerous assumptions. Although C‒O can provide a reasonable infrared intensity, Raman spectroscopy occurs most intensely for symmetric vibrations such as C‒C and C=C. Infrared spectroscopy is allegedly more suited to polar groups in which a displacement of charge occurs between electropositive and electronegative atoms such as N‒H and O‒H [35].

#### 3.1.3. In-situ Scanning Probe Microscopy (SPM)

**Figure 3:**
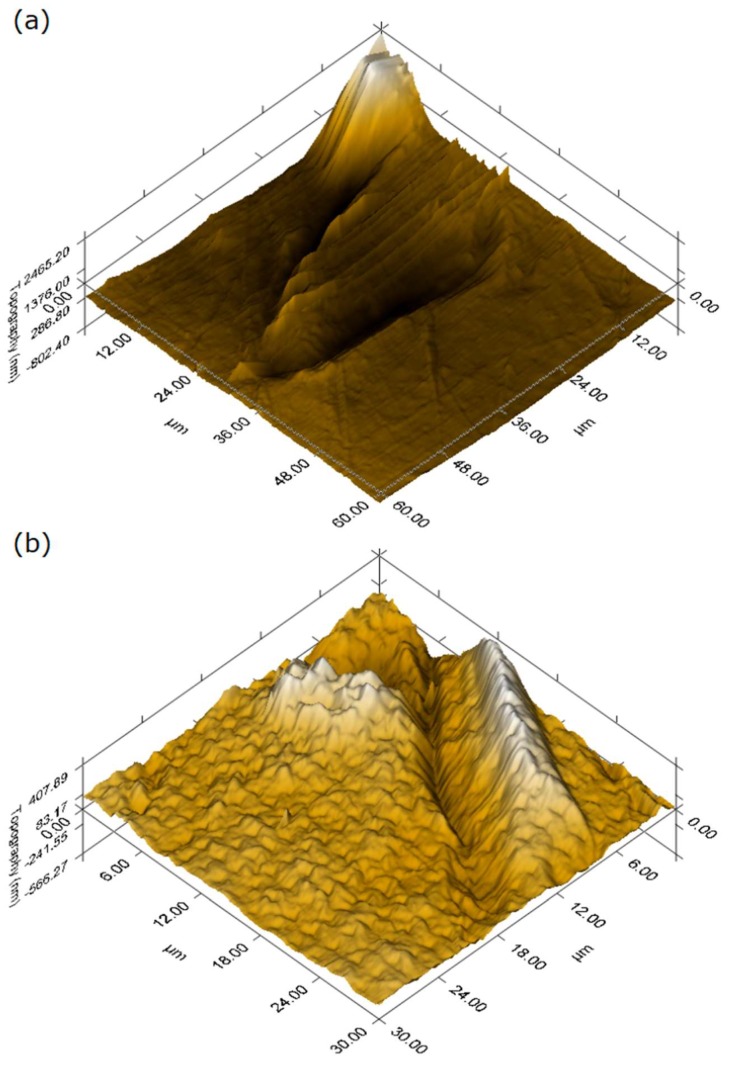
In-situ scanning probe microscopy (SPM) imaging of PVDF–SSPF composites. (**a**) 3D direct measurement of surface topography of up to 60 µm; (**b**) high-resolution topography mapping with another length scale of up to 30 µm.

Using in-situ scanning probe microscopy (SPM), two 3D presentations were obtained from two different areas. In this section, the structure and surface topography of PVDF–SSPF are presented to further provide information on the behavior and structure of specimens. Figure 3 displays the in-situ scanning probe microscopy imaging of composites. 

The images confirmed favorable compaction and excellent bonding between the fiber and matrix. The consolidation of PVDF–SSPF is clearly shown with some regular and minor protruding on the topographical surface of specimens. This can be considered as slight roughness which occurs during the fabrication process, especially when hot compression is applied. Eliminating the protruding sections using surface-smoothing methods is beneficial. The matrix surface can be treated and prepared for lamination if required. In addition to favorable structural properties of both materials, the outstanding interfacial bonding between the fiber and matrix can also be attributed to the utilization of short fiber. In various research works, improvements in certain properties were observed in short fiber. No significant effect in flexural strength and density was observed in composites when length increased [36]. Hence, the use of short fiber contributed to the interfacial bonding of the fiber matrix with stress-transfer efficiency, resulting in a homogenous structure within the composites. The light-colored areas may represent a high modulus since a correlation can be found between the fiber’s morphology and high modulus. The fiber seems to induce the mechanical compound of the matrix composite [37]. 

### 3.2. Thermal Properties

#### 3.2.1. The Dynamic Mechanical Analysis (DMA)

**Figure 4:**
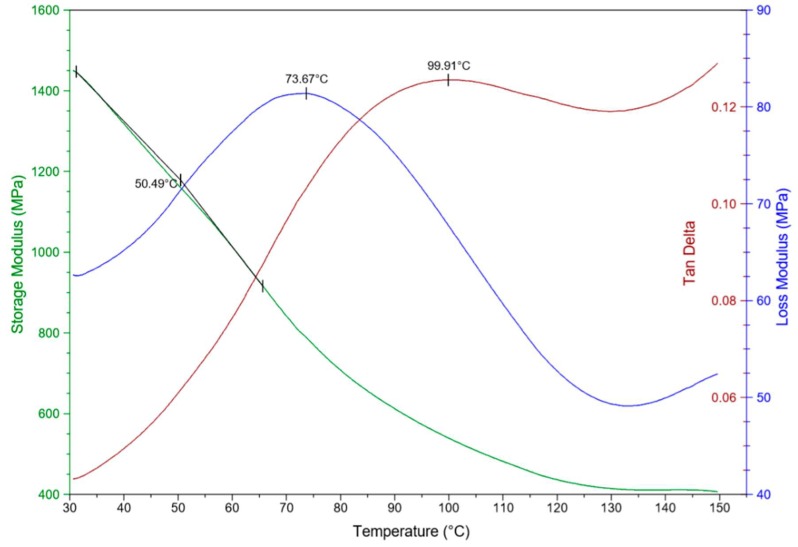
Typical DMA plot analysis presenting the impact on storage and loss modulus.

The storage modulus curves provide valuable insights to further comprehend the stiffness of composites as a function of temperature. The curves can also be useful in evaluating the molecular basis of mechanical properties as they might behave sensitively to structural changes, e.g., fiber matrix interfacial bonding, degree of cross-linking, and molecular weight. Under certain circumstances, the loss modulus can increase with fiber loading. The maximum heat dissipation occurs at the temperature where E” is at its maximum value [38]. The storage and loss modulus are given in Figure 4.

The increase of storage modulus can be interpreted by the addition or increase of SSPF fiber in the matrix composites. When the temperature rises, the composites may become unsteady and lose close packing. Decent performance in the storage modulus can be observed, which may be due to the thermal stability shown. The proportional storage and loss modulus in the composites displayed an overall acceptable behavior and this can be explained by the increase of fiber content and the homogenous distribution of fiber in the matrix. However, the interference of neighboring chains and the higher restriction imposed by former fibers can also be considered as factors that contribute to enhanced storage modulus [39]. The mechanical behavior of PVDF–SSPF composites was further investigated. The viscoelastic properties and the effects of fiber loading on the rheological properties of composites were precisely evaluated. To compare with previous results, the storage modulus of natural fiber composites (NFCs) was analyzed through various works, different results were obtained. In the research by Bachtiar et al. [40], the effects of temperature on the storage modulus, loss modulus, and mechanical damping (tan d) for untreated SSPF reinforced with high impact polystyrene (HIPS) composites were explored. It was discovered that the storage modulus increased with the increase of fiber content, excluding 10% fiber content. It was also asserted that the addition of fiber leads to better stability in composites. From the (tan d) results, the fiber composites had smaller energy dissipation coefficients than the pure HIPS matrix [40]. Jacob et al. [38] investigated the dynamic mechanical properties of sisal/oil palm hybrid fiber reinforced with natural rubber composites and concluded that the storage modulus increases when increasing the fiber loading in the matrix. The same study explained that the reinforcement imparted by the fibers led to a strong and stiff interface in composites [38]. The study of Kumar et al. [41] examined untreated and treated coconut sheath fiber reinforced epoxy composites. The study discovered increased storage modulus in the treated fiber and higher adhesion in treated composites compared to untreated ones [41]. 

#### 3.2.2. The Thermomechanical Analysis (TMA)

**Figure 5:**
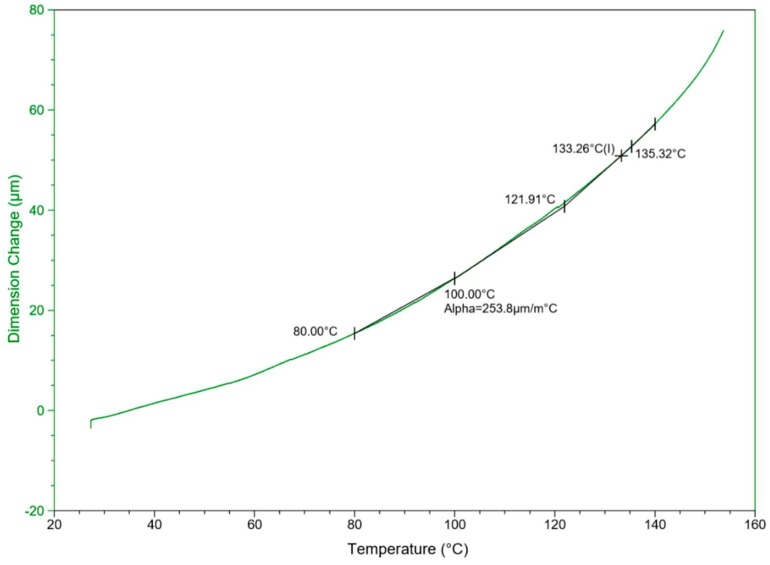
Typical thermomechanical curve for PVDF–SSPF showing the linear expansions based on temperature *T* (°C) and dimension change (µm).

The TMA analysis is an essential method for measuring various properties depending on temperature and time. The analysis offers accurate evaluations of dimensional changes by defining the coefficient of thermal expansion (CTE) and assessing the changes caused by altered temperatures. Figure 5 provides information on the overall performance of PVDF–SSPF composites in the thermomechanical analysis. 

The TMA dimensional change and temperature curves display the aggregate behavior of thermally processed specimens. In the evaluation of the normalized value of CTE expansion or dimensional changes, the behavior of composites presented exceptional thermal stability and mechanical strength as well as adequate compatibility of fiber and polymer for outdoor applications. The CTE values were in the temperature range of 20–160 °C with a constant heating rate. At 100 °C, Alpha was determined as 253.8 µm/m °C. The thermal expansion occurred as a natural correspondence to the thermal increase causing the dimensional change in the composites. Maintaining suitable thermal expansion reflects on the composites’ satisfactory interfacial properties. The measurement of the thermal expansion coefficient (TEC) can be beneficial for determining the thermal stresses and dimensional changes caused by thermal variation. However, the TEC in natural fiber composites (NFCs) may appear lower than that of pure polymer structures. Lower TEC can be useful in minimizing thermo-dimensional changes, especially in composites that are subjected to temperature changes in either the fabrication or utilization processes [42].

#### 3.2.3. The Thermogravimetric Analysis (TGA) 

**Figure 6:**
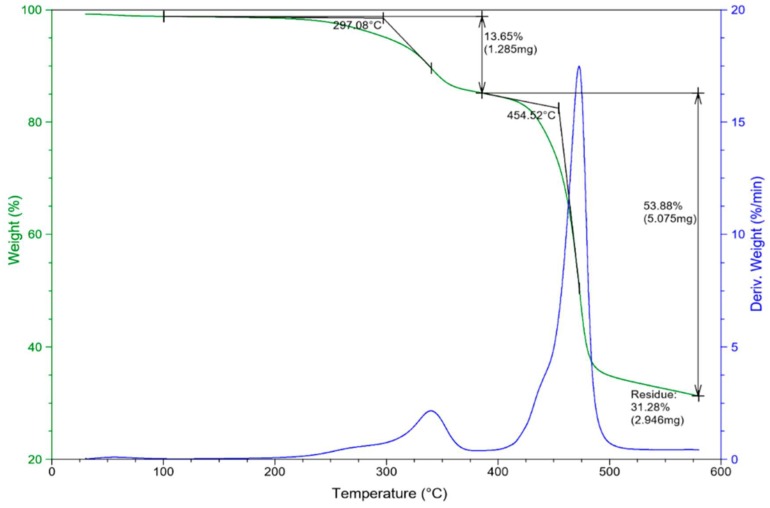
Thermogravimetric plot analysis of PVDF–SSPF composites.

The test of thermogravimetric analysis (TGA) is an essential method that determines the thermal degradation of composites and characterizes their thermal behavior, providing extensive information regarding thermal stability. The process entails a continuous evaluation of changes occurring on the material’s weight under constant increase of temperature within controlled measurements. Figure 6 provides the thermogravimetric plot analysis, reflecting on the performance of composites throughout the test.

Thermal behavior has a significant impact on the overall performance of the fiber matrix composites. Therefore, it is necessary to understand their behavior before processing or considering any manufacturing procedures. Low thermal stability is considered as one of the limiting factors for utilizing natural fiber materials in biocomposites [8]. The weight loss and temperature indicate good thermal stability in the composite despite the initial degradation of fiber. Technically, there are three main phases in the thermal decomposition of fibers: The correspondence of moisture evaporation, hemicellulose, cellulose, and lignin degradation, and finally, residues [43]. The decomposition of composites is estimated to range between 30 °C to 600 °C. The curves display composites with a reduction in mass loss, which occurs as a function of temperature. The composites withstood high temperature due to favorable thermal properties of both the fiber and polymer matrix. The TGA analysis is useful to detect the presence of functional groups in composite materials [43]. Rashid et al. [43] employed the TGA technique on treated and untreated SPF. Untreated SPF displayed significant results and higher stability compared to treated fiber. The fiber of sugar palm has a very high chemical content of cellulose that reaches 43.87%. This value contributes to higher thermal stability and strength within the fiber. In hybrid composites, the loading of fiber may slightly reduce thermal stability when applying the TGA test. This can be due to the evaporation of moisture or the decomposition of the natural fiber’s major constituents, hemicelluloses, cellulose, and lignin [29].

#### 3.2.4. The Differential Scanning Calorimetry (DSC)

**Figure 7:**
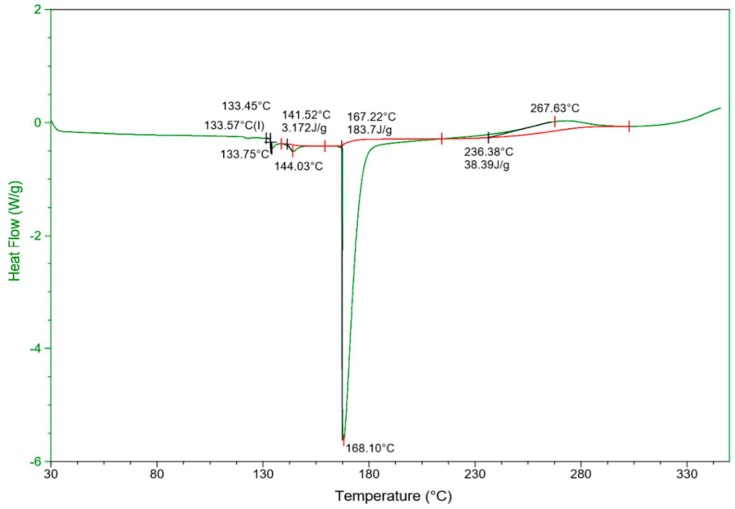
Differential scanning calorimetry (DSC) result on the performance of composites, considering heat flow versus temperature.

The test of differential scanning calorimetry (DSC) was accomplished to determine the difference in heat flow, to establish the required amount to increase temperature, and to compare the thermal behavior of composites. Figure 7 provides the DSC scan of PVDF–SSPF, displaying the overall performance of composites.

Based on Figure 7, the heating rate ranged between −6 and 2 W/g and processed under increasing temperatures, from 30 °C to 330 °C. The DSC analysis of PVDF–SSPF revealed a single melting peak with a maximum endothermic peak (energy absorbed) at 168.10 °C. The development of crystallinity is linked to the exothermic peak (energy released) attained by applying the DSC test and integrated to calculate the mass fraction of crystallinity. See Table 1 for further numerical presentation. The maximum peak integration was 144.03 °C, 168.10 °C, and 267.63 °C, respectively, and automatically concluded at 302.68 °C. This indicates overall satisfactory DSC properties in the composites, withstanding constant increasing heat and resisting degradation at low temperatures. The midpoint of glass transition was indicated at 133.57 °C, with height W/g of 0.1604 and delta Cp of 0.9735 J/(g·°C). However, in correlation with another accomplished thermal testing, the degradation of PVDF–SSPF composites’ mechanical properties occurred under increased temperature. When comparing the overall performance of composites, their thermal stability is evident by the overall performance of specimens and the rate of mass reduction in the composites under high temperature. A decrease in mechanical properties was also observed. This can be attributed to the excessive heat applied which induced thermal degradation and caused a decrease in the specimens’ weight. In natural fibers, a decrease in the position of the peak can be justified by the increase of amorphous cellulose and decrease of cellulose crystallite length. The mass loss during the DSC analysis was attributed to degradation which took place during the test. Wider molecular size distribution and weight of molecules may cause a shift in peak, therefore, the endothermic peak of the fusion behavior will impact the degradation reaction in the exothermic peak [44].

### 3.3. Technical Properties

#### 3.3.1. Moisture Content

The moisture content test is an essential process to determine the moisture in fabricated specimens and to understand the technical behavior of these composites. The composites presented very little moisture after being immersed in water for 1500 h. Alaaeddin et al. [37] examined the moisture content of similar specimens without water immersion. After specimens were dried in the oven for 24 h, they exhibited extremely low moisture content. Therefore, a prolonged immersion test was accomplished to investigate the moisture content in composites under excessive exposure to water. Since these specimens were prepared for outdoor applications, it is necessary to assess their technical properties in harsh and unusual environments. Table 2 provides information on the aggregate test of moisture content. 

The moisture absorption behavior of SPF reinforced composites is based on two main quantities. Firstly, the duration of the transient linked to the sample thickness and the diffusion coefficient known as diffusivity (the characteristic of the transport rate of water molecules in composites). Secondly, the equilibrium moisture content in which a characterization occurs to the composite’s affinity for hydrophilicity.

#### 3.3.2. Water Absorption

Under successive testing phases, the water absorption analysis was accomplished based on multiple readings to acquire an extensive understanding of the specimens’ absorption behavior. Table 3 provides further information on the obtained results. 

**Figure 8:**
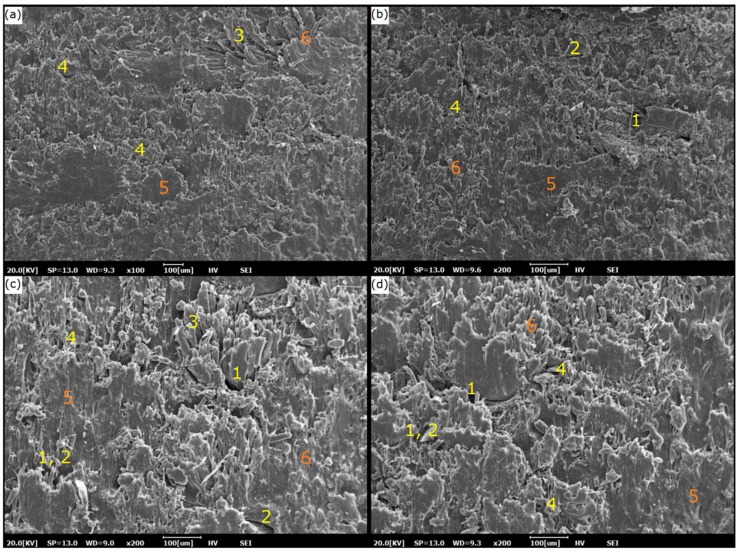
Cross-sectional SEM image of immersed composites. (**a**,**b**) Two cross-sectional views from the first perspective. (**c**,**d**) Two cross-sectional views from the second perspective. (**1**) Void, (**2**) fiber matrix debonding, (**3**) matrix disarrangement, (**4**) porosity, (**5**) good surface adhesion, (**6**) good matrix bonding.

Besides the reduction of void occurrence and the hindered diffusion of water through composites, strong interfacial bonding between the fiber and matrix may have contributed to the lower uptake of water [45]. A clear decline in water absorption can be observed. The specimens were in good condition after consecutive immersion of 2000 h. However, due to the natural characteristics of SSPF, water uptake constantly increased, reaching a steady value at saturation point. Despite that, the natural characteristic of SSPF can also translate to low water absorption in composites since SPF is known for its durability and resistance to seawater [28]. This is due to the cellulose and lignin which contain free hydroxyl group within their structure. Figure 8 provides an SEM image of the immersed specimens, showing a cross-sectional view of the composite structure after being immersed in distilled water. Saturation in water mass concentricity can be identified as a constant that can be reached when the interaction between the composites and water is reversible [46]. Water absorption of hybrid composite decreases when increasing the load of SPF in the matrix composite. This was also found in the moisture content analysis. Reduced water uptake in composites can be attributed to lower hydrophilic behavior of SPF [29].

Good interfacial bonding and matrix adhesion can be attributed to the favorable properties of the fiber and matrix structures. The incorporation of SSPF contributed to the decline of water absorption. In addition, the homogenous distribution of fiber and the rigid structure led to the overall performance of composites under various weather conditions. In some studies, it has been reported that water absorption shrank in biocomposites with increased fiber loading due to obstructed absorption instigated by the fibers and better matrix-fiber interfacial bonding. Additionally, the fiber loading was found to enhance the composites’ thermal stability, prompting the anticipation of further applications for these biocomposites [21]. 

#### 3.3.3. Thickness Swelling 

When specimens are immersed in water for 1500 h, a weight gain and deformation may occur due to the water absorption phenomena. Three-dimensional testing was conducted on the specimens. The composites exhibited excellent dimensional stability. Table 4 shows the overall performance of specimens before and after immersion.

Photovoltaic module backsheets can remarkably contribute to the overall yield as well as the module’s lifespan. Hence, differences can be observed in module power due to the utilization of various types of backsheets. Even with similar global reflectance, the angular components of reflected light (angular response), light reflectance, performance against the penetration of water vapor and moisture from the atmosphere, as well as cell-to-module losses are important factors to enhance module efficiency [47]. PVDF–SSPF is characterized according to its materials and potential performance in photovoltaic applications. The investigated properties of composites revealed a homogenous structure between the fiber and polymer matrix. The composites performed adequately when exposed to water and displayed high resistance to water absorption and moisture content. The overall evaluation proves that composites are suitable candidates for novel solar modules. The composites further exhibited reasonable optical performance and presented good thermal stability. However, it is recommended to directly examine composites on photovoltaic modules to investigate their direct impact on these modules. More relevant information can be obtained on the performance of composites under various weather conditions so as to detect early predictors of backsheet failure. Fabricating these composites with photovoltaic modules will contribute to the understanding of their behavior, especially when conducting comparative life-cycle assessments. Investigating environmental impacts, optical performance, thermal stability, their direct and indirect influences on modules, as well as reusing possibilities and comparing obtained results to other backsheets are necessary. Since advancements in photovoltaic backsheets led to a number of novel backsheet materials, introduced backsheets must prevent moisture in outdoor and climate chambers from entering the module. They must fit the specific module requirements for better performance and higher efficiency [48]. 

## 4. Conclusions

This work introduces novel, lightweight PVDF–SSPF composites for photovoltaic module backsheets. Their optical, thermal, and technical properties were investigated. The composites revealed great compatibility, thermal stability, and excellent interfacial bonding between the fiber and matrix components. It is, therefore, concluded that:

- In the optical testing, exposing the specimens to water leads to slight changes in molecular interactions in which a minor shift in wavenumber can be observed. The FTIR spectra demonstrated the existence of specific chemical constituents in the PVDF–SSPF composites. Good stability in absorbance and transmittance values was observed.
- The Raman spectra characterized the fiber and polymer and supported fibers based on their compositions. Minimized peaks were observed for immersed specimens. This was due to the water content within the composites. The optical analyses provide strong evidence that the fiber did interact with the polymer matrix. This was confirmed by the in-situ SPM imaging.
- The composites exhibited excellent thermal stability. The proportional storage and loss modulus of DMA were examined. Typical dimensional changes and CTE expansion under constant heating rate in TMA were observed. Thermal expansion occurred as a natural correspondence to thermal increase, causing dimensional changes in the composites. In TGA, the weight loss and temperature indicated exceptional thermal stability despite the initial degradation of fiber. The composites withstood high temperature, causing mass loss to occur as a result.
- The DSC analysis revealed a single melting with a maximum endothermic peak at 168.10 °C. The maximum peak integration was addressed. The composites responded to constantly increasing heat and resisted degradation at low temperatures. The degradation of PVDF–SSPF composites’ mechanical properties occurred under increased temperature. Thermal stability was proven by the overall performance of specimens and the rate of mass reduction in composites under high temperature.
- The application of short fiber contributed to interfacial bonding of the fiber matrix with stress-transfer efficiency and resulted in a homogenous structure within the composites. The composites’ excellent technical properties were addressed while considering prolonged water immersions.

## Figures and Tables

**Table 1:** A comprehensive process analysis on the thermal procedure with numerical results.

**The Dynamic Mechanical Analysis (DMA)**																				
**Onset Point**											**Signal Maximum**									
**Start °C**			**Stop °C**		**Onset °C**	**Onset Y MPa/Storage Modulus**					**Start °C**			**Stop °C**			**Maximum °C**		**-**	
31.21			65.67		50.49	1179					30.69			129.68			99.91		Tan Delta	
											30.69			129.68			73.67		Loss Modulus	
**The Thermomechanical Analysis (TMA)**																				
**Step Transition/Signal (Dimension Change)**											**Alpha X1 to X2 / Signal (Dimension Change)**									
**Onset °C**			**Midpoint (I) °C**		**End °C**	**Height µm**					**Start °C**			**Manual Onset °C**			**Alpha µm/(m·°C)**			
121.91			133.26		135.32	30.87					80.00			100.00			253.84			
**The Thermogravimetric Analysis (TGA)**																				
**Onset Point**						**Weight Change**										**Residue**				
**Start °C**	**Stop °C**		**Onset °C**		**Onset Y %**	**Start °C**		**Stop °C**		**Weight mg**			**Weight %**			**Temperature °C**		**Weight mg**		**Weight %**
100.23	340.17		297.08		98.55	100.23		385.61		1.285			13.65			579.94		2.946		31.28
385.73	472.90		454.52		82.49	385.73		579.38		5.075			53.88							
**The Differential Scanning Calorimetry (DSC)**																				
**Peak Integration**												**Glass Transition**								
**Start °C**		**Onset °C**		**Maximum °C**			**Stop °C**		**Area J/g**			**Onset °C**		**Midpoint (I) °C**	**End °C**		**Height W/g**		**Delta Cp J/(g·°C)**	
138.64		141.52		144.03			159.20		3.172			133.45		133.57	133.75		0.1604		0.9735	
159.20		167.22		168.10			214.47		183.7											
214.47		236.38		267.63			302.68		38.39											

**Table 2:** Overall evaluation of moisture content.

SPs	Weight/g	Room Temperature °C	Test Temperature °C	Moisture (%)	Total Time/s	Immersion Time/h
1–5	2.0±	27±	120	0.79	202	1500

**Table 3:** Summary of water absorption test results of PVDF–SSPF composites.

**SPs**	**Weight/g**	**300 h**	**Water Absorption (%)**	**1000 h**	**Water Absorption (%)**
SP1	0.731	0.771	5.471956224	0.779	6.566347469
SP2	0.712	0.723	1.54494382	0.725	1.825842697
SP3	0.770	0.783	1.688311688	0.787	2.207792208
SP4	0.719	0.732	1.808066759	0.736	2.364394993
SP5	0.737	0.754	2.306648575	0.759	2.985074627
**Mean**	**0.7338**	**0.7526**	**2.562005996**	**0.7572**	**3.188879804**
**SPs**	**Weight/g**	**1500 h**	**Water Absorption (%)**	**2000 h**	**Water Absorption (%)**
SP1	0.731	0.786	7.52393981	0.787	7.66073871
SP2	0.712	0.728	2.24719101	0.733	2.9494382
SP3	0.770	0.79	2.5974026	0.794	3.11688312
SP4	0.719	0.739	2.78164117	0.742	3.19888734
SP5	0.737	0.763	3.52781547	0.767	4.07055631
**Mean**	**0.7338**	**0.7612**	**3.73398746**	**0.7646**	**4.19732897**

**Table 4:** Summary of thickness swelling test results for PVDF–SSPF composites.

SPs	Room Temperature °C	Water Temp. °C	3-points Averaged Dimensions (mm)	3-points Averaged Swellings (mm)	1500 h Thickness Swelling (%)
SP1	27 ± 0.5	25.4 ± 0.01	2.09	2.15	2.86 ± 0.01
SP2	27 ± 0.5	25.4 ± 0.01	2.14	2.19	2.64 ± 0.01
SP3	27 ± 0.5	25.4 ± 0.01	2.01	2.06	2.14 ± 0.01
SP4	27 ± 0.5	25.4 ± 0.01	2.05	2.12	3.24 ± 0.01
SP5	27 ± 0.5	25.4 ± 0.01	1.98	2.02	2.18 ± 0.01
Mean			2.05	2.11	2.62 ± 0.01
